# Supplementary material for: Engineering rice Nramp5 modifies cadmium and manganese uptake selectivity using yeast assay system
Source: Front Plant Sci. 2024 Nov 20;15:1482099. doi: 10.3389/fpls.2024.1482099 (PMC11614607; doi:10.3389/fpls.2024.1482099)
Supplement: Supplementary file 1 [file DataSheet1.pdf]

Supplementary Material

Engineering of rice Nramp5 modifies cadmium and manganese uptake selectivity using yeast assay system

Junji Inoue, Takamasa Teramoto, Tomohiko Kazama, Takahiro Nakamura*

Faculty of Agriculture, Kyushu University, Fukuoka, Japan

*** Correspondence:**

Takahiro Nakamura

tnaka@agr.kyushu-u.ac.jp

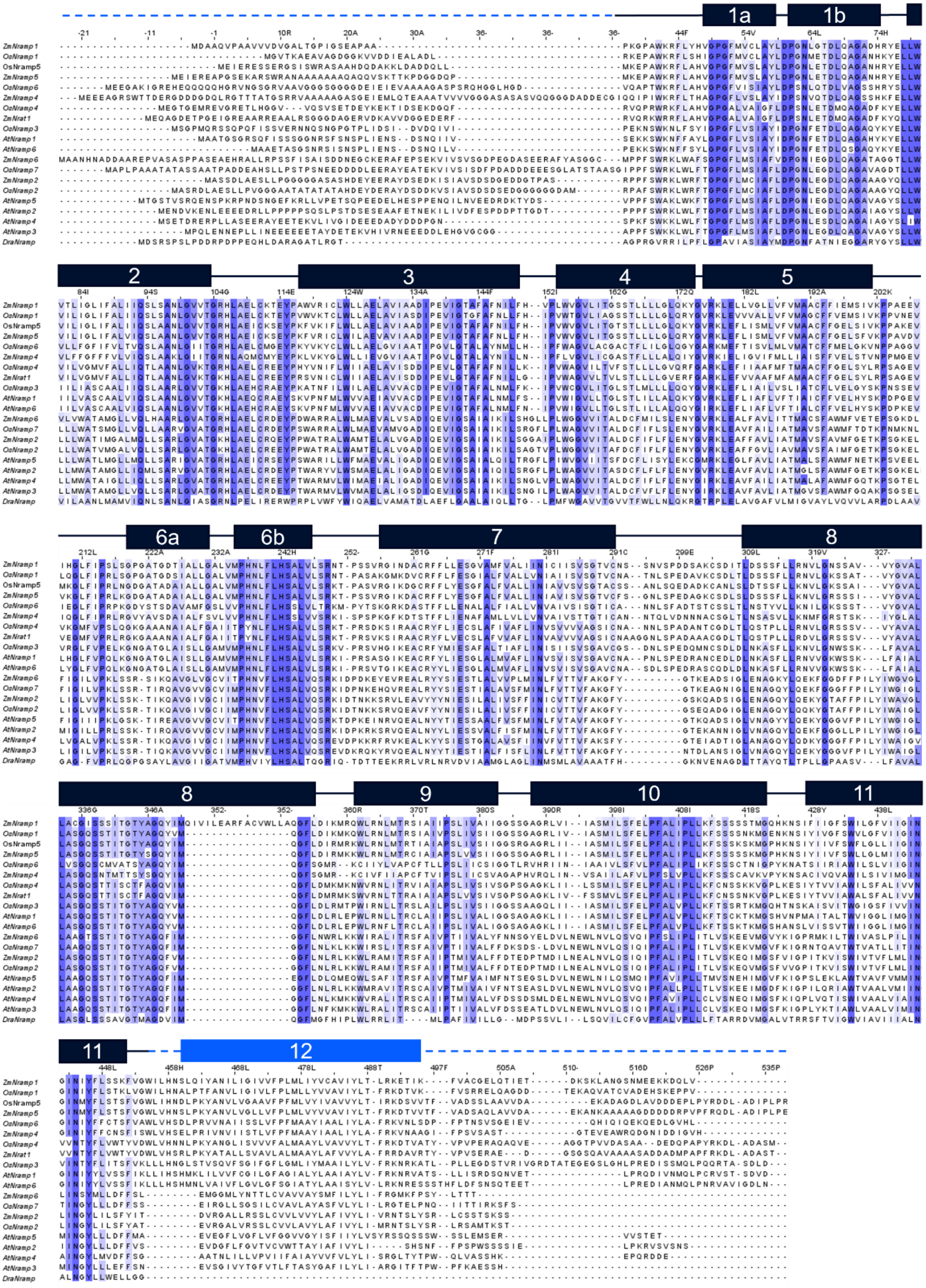

Supplementary Figure 1. Amino acid sequences of Nramp proteins. Amino acid sequences of 20 Nramps shown with numbers corresponding to OsNramp5. The amino acid conservation is shown in blue. The transmembrane region is schematically represented.

A

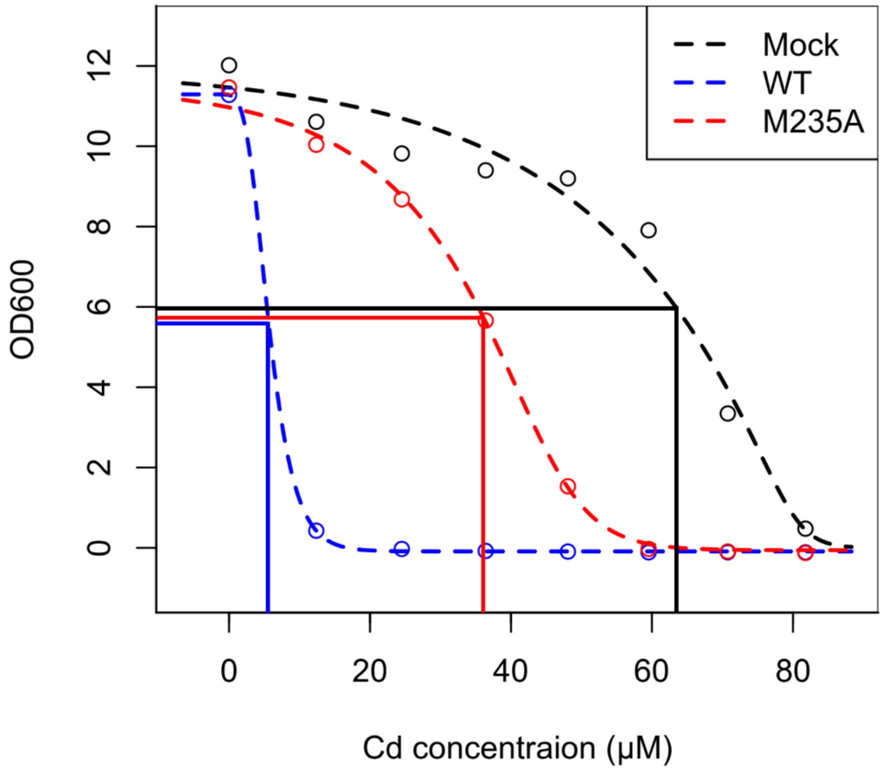

B

	IC_{50}	1 / IC_{50}	Relative Cd uptake (%)
Mock	63.5	0.016	0.0
WT	5.6	0.178	100.0
M235A	36.1	0.028	7.4

Supplementary Figure 2. Example of estimation of the metal uptake efficiency. (A) Cd-sensitive strain (*Δycf1*) containing the plasmid (WT, M235A, or EV) was incubated in various Cd concentrations (0, 20, 40, 60, or 80 μM). The growth was measured by the OD600 and the maximum likelihood curves are depicted to estimate the IC_{50}. **(B)** Metal uptake efficiency estimated as the inversed score of IC_{50} (1/ IC_{50}) with adjustment of the score of WT and EV as 100 and 0, respectively.

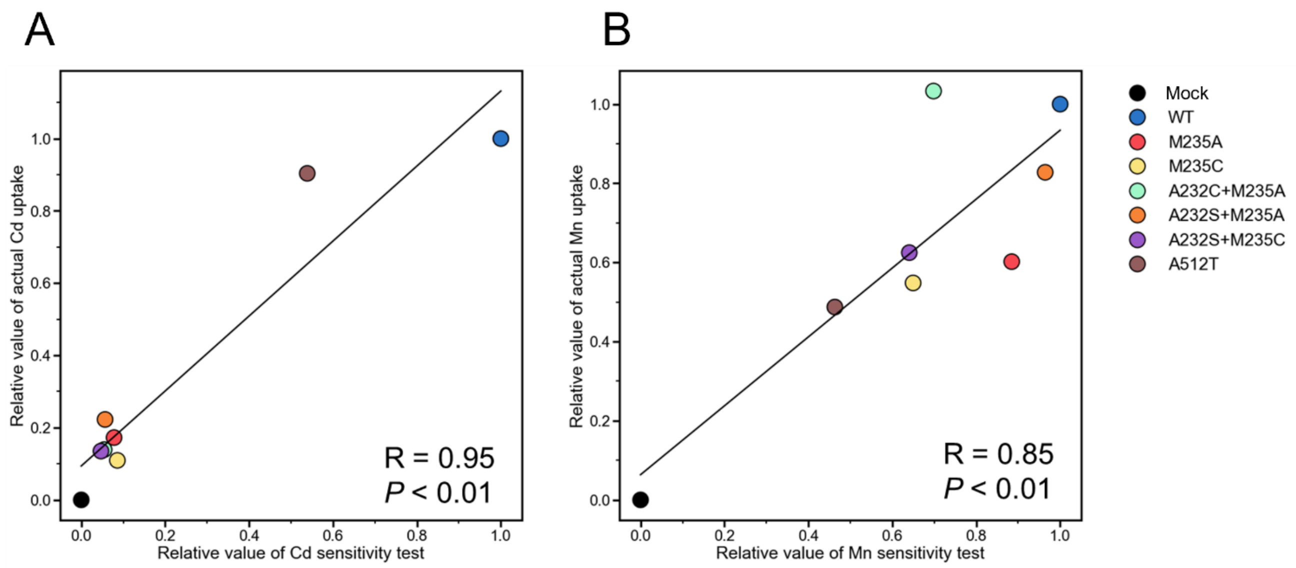

Supplementary Figure 3. Compatibilities between the result of the yeast assay system and ICP-OES. (A) Relative Cd uptake value obtained by the yeast assay system (Table 2) and scatter plot of the direct measurement using ICP-OES (Table 3). Pearson's correlation coefficient R and the *P* values are shown to estimate the compatibility. **(B)** Relative Mn uptake values obtained from the two methodologies in (A).

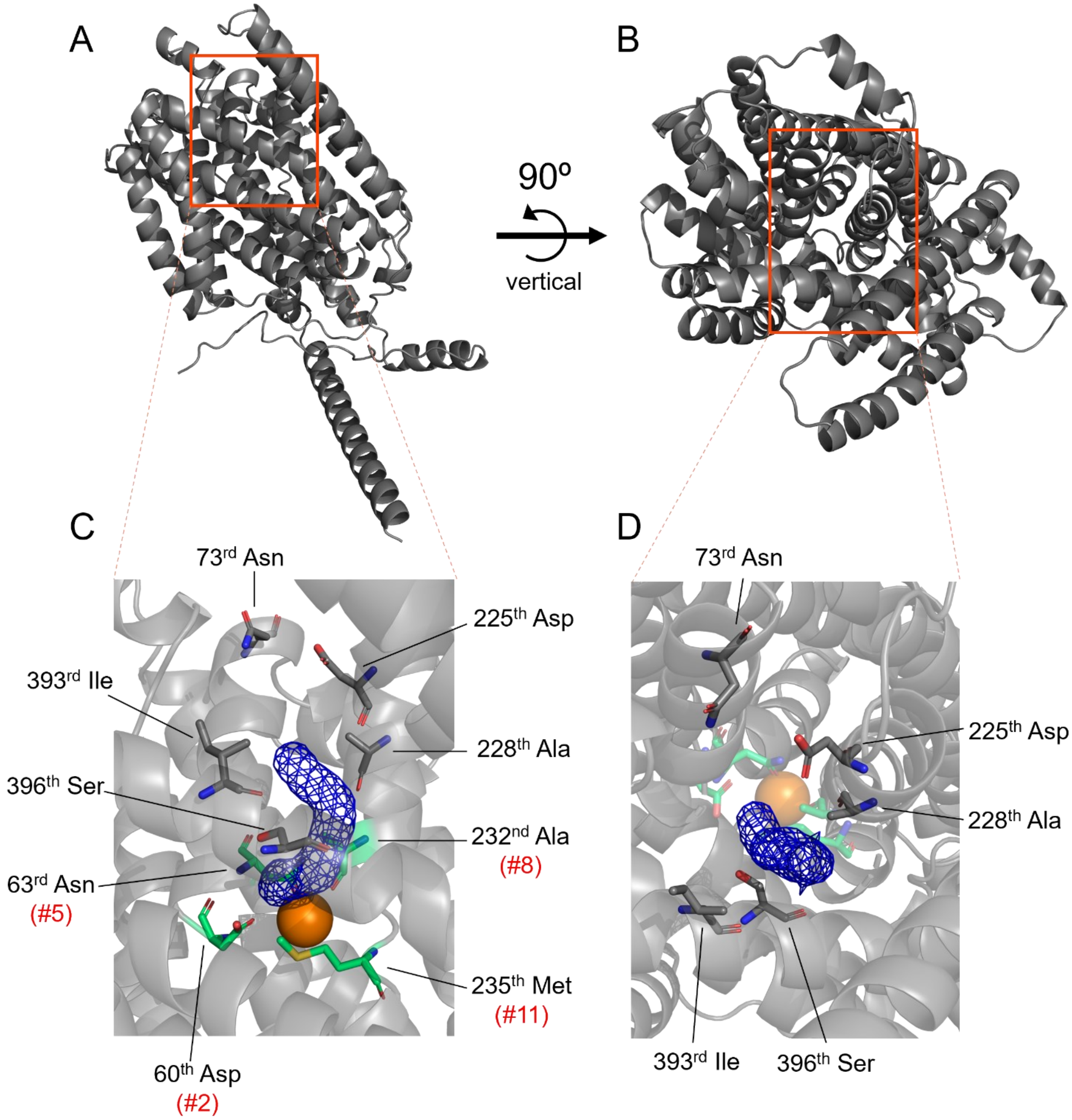

Supplementary Figure 4. Predicted Mn transport pathway of OsNramp5. Predicted structure of OsNramp5 viewed from the **(A)** membrane side and **(B)** extracellular metal entry side. **(C)** Predicted transport tunnel from the membrane side. The transport tunnel and Mn^{2+} ions are shown by blue mesh and orange spheres, respectively. The key residues for the metal uptake efficiency (#2, #5, #8, and #11) are shown by green sticks, together with the amino acid residues that have been demonstrated to be involved in the metal uptake efficiency and selectivity of other Nramps. **(D)** Predicted transport tunnel from the extracellular or metal entry side shown as in (C).

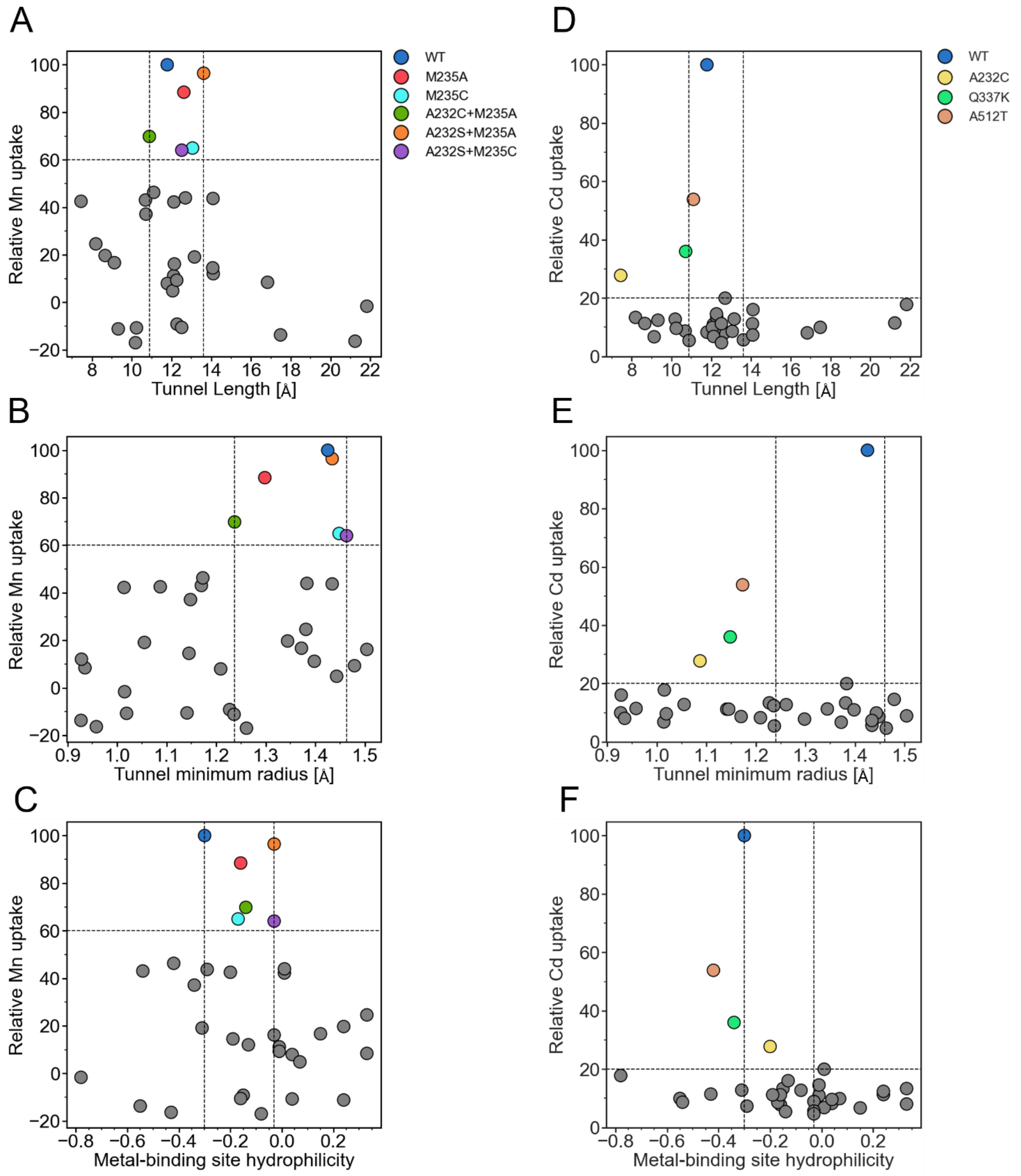

Supplementary Figure 5. Relationship between the relative metal uptake efficiency and spatial/chemical properties of the predicted 3D structure. (A) Relative Mn uptake of the OsNramp5 and 33mutants in this study (Table 1 and 2) scatter-plotted according to the tunnel length of the predicted structure. The proteins that showed more than 60% Mn uptake efficiency were positioned in the tunnel length between 10.88 and 13.61 Å. Scatter plots of relative Mn uptake and (B) tunnel minimum radius and (C) metal-binding site hydrophilicity are shown, as well as for relative Cd uptake (D, E, and F).

Supplementary Table 1. Nramp genes for the phylogenetic analysis

Gene	Accession	Locus
AtNramp1	NP_178198	AT1G80830
AtNramp2	NP_175157	AT1G47240
AtNramp3	NP_179896	AT2G23150
AtNramp4	NP_201534	AT5G67330
AtNramp5	NP_193614	AT4G18790
AtNramp6	NP_173048	AT1G15960
OsNramp1	NP_001389971	Os07g0258400
OsNramp2	NP_001404287	Os03g0208500
OsNramp3	NP_001411161	Os06g0676000
OsNramp4	NP_001396197	Os02g0131800
OsNramp5	NP_001389970	Os07g0257200
OsNramp6	NP_001393044	Os01g0503400
OsNramp7	NP_001410524	Os12g0581600
ZmNramp1	XP_008670084	GRMZM2G366919
ZmNramp2	NP_001150280	GRMZM2G178190
ZmNramp4	XP_008670762	GRMZM2G322844
ZmNramp5	NP_001354866	GRMZM2G147560
ZmNramp6	XP_008665146	GRMZM2G025680
ZmNrat1	NP_001334019	GRMZM2G168747
ScaNramp	WP_002452964	-
DraNramp	Q9RTP8	-

Supplementary Table 2. Sequence of primers

Primer name	Forward(5'-3')	Reverse(3'-5')
OsNramp5_GA	AAAAATATACCCCAGCCATGGAGATTGAGAGAGAGAGC	GAAGAAGTCCAAAGCTGACACCCTTGTCGATCGATC
M235A	CTTGTCGCGCCCCACAATCTGTTCTTG	GTGGGGCGCGACAAGAGCTCCGAGGAG
M235C	CTTGTCTGCCCCCACAATCTGTTCTTG	GTGGGGGCAGACAAGAGCTCCGAGGAG
M235D	CTTGTCGACCCCCACAATCTGTTCTTG	GTGGGGGTCGACAAGAGCTCCGAGGAG
M235E	CTTGTCGAGCCCCACAATCTGTTCTTG	GTGGGGCTCGACAAGAGCTCCGAGGAG
M235F	CTTGTCTTCCCCCACAATCTGTTCTTG	GTGGGGGAAGACAAGAGCTCCGAGGAG
M235G	CTTGTCGGGCCCCACAATCTGTTCTTG	GTGGGGCCCGACAAGAGCTCCGAGGAG
M235H	CTTGTCCATCCCCACAATCTGTTCTTG	GTGGGGATGGACAAGAGCTCCGAGGAG
M235I	CTTGTCATACCCCACAATCTGTTCTTG	GTGGGGTATGACAAGAGCTCCGAGGAG
M235K	CTTGTCAAGCCCCACAATCTGTTCTTG	GTGGGGCTTGACAAGAGCTCCGAGGAG
M235L	CTTGTCCTGCCCCACAATCTGTTCTTG	GTGGGGCAGGACAAGAGCTCCGAGGAG
M235N	CTTGTCAATCCCCACAATCTGTTCTTG	GTGGGGATTGACAAGAGCTCCGAGGAG
M235P	CTTGTCCCGCCCCACAATCTGTTCTTG	GTGGGGCGGGACAAGAGCTCCGAGGAG
M235Q	CTTGTCCAGCCCCACAATCTGTTCTTG	GTGGGGCTGGACAAGAGCTCCGAGGAG
M235R	CTTGTCAGGCCCCACAATCTGTTCTTG	GTGGGGCCTGACAAGAGCTCCGAGGAG
M235S	CTTGTCAGTCCCCACAATCTGTTCTTG	GTGGGGACTGACAAGAGCTCCGAGGAG
M235T	CTTGTCACGCCCCACAATCTGTTCTTG	GTGGGGCGTGACAAGAGCTCCGAGGAG
M235V	CTTGTCGTGCCCCACAATCTGTTCTTG	GTGGGGCACGACAAGAGCTCCGAGGAG
M235W	CTTGTCTGGCCCCACAATCTGTTCTTG	GTGGGGCCAGACAAGAGCTCCGAGGAG
M235Y	CTTGTCTACCCCCACAATCTGTTCTTG	GTGGGGGTAGACAAGAGCTCCGAGGAG
A232S	CTCGGAAGTCTTGTCATGCCCCACAATCTGTTC	GACAAGACTTCCGAGGAGGGCAATGGCGTCGGC
A232V	CTCGGAGTGCTTGTCATGCCCCACAATCTGTTC	GACAAGCACTCCGAGGAGGGCAATGGCGTCGGC
A232C	CTCGGATGCCTTGTCATGCCCCACAATCTGTTC	GACAAGGCATCCGAGGAGGGCAATGGCGTCGGC
A232M	CTCGGAATGCTTGTCATGCCCCACAATCTGTTC	GACAAGCATTCCGAGGAGGGCAATGGCGTCGGC
A232S+M235A	GGAAGTCTTGTCGCGCCCCACAATCTGTTC	GGGCGCGACAAGACTTCCGAGGAGGGCAAT
A232V+M235A	GGAGTGCTTGTCGCGCCCCACAATCTGTTC	GGGCGCGACAAGCACTCCGAGGAGGGCAAT
A232C+M235A	GGATGCCTTGTCGCGCCCCACAATCTGTTC	GGGCGCGACAAGGCATCCGAGGAGGGCAAT
A232M+M235A	GGAATGCTTGTCGCGCCCCACAATCTGTTC	GGGCGCGACAAGCATTCCGAGGAGGGCAAT
A232S+M235C	GGAAGTCTTGTCTGCCCCCACAATCTGTTC	GGGGCAGACAAGACTTCCGAGGAGGGCAAT
A232V+M235C	GGAGTGCTTGTCTGCCCCCACAATCTGTTC	GGGGCAGACAAGCACTCCGAGGAGGGCAAT
A232C+M235C	GGATGCCTTGTCTGCCCCCACAATCTGTTC	GGGGCAGACAAGGCATCCGAGGAGGGCAAT
A232M+M235C	GGAATGCTTGTCTGCCCCCACAATCTGTTC	GGGGCAGACAAGCATTCCGAGGAGGGCAAT
Q337K	TCTGGGAAGAGCTCCACTATTACCGGC	GGAGCTCTTCCCAGATGCCAACAGTGC
A512T	GAGAAGACCGACGCCGGCGACCTCGCC	GGCGTCGGTCTTCTCGGCGTCGACGAC

Supplementary Table 3. Direct measurement of Co, Cu, Fe, and Zn contents for OsNramp5 mutants.

	Co in yeast (ng/mg)	±	SD		Cu in yeast (ng/mg)	±	SD		Fe in yeast (ng/mg)	±	SD	Zn in yeast (ng/mg)	±	SD
Mock	16.1	±	2.2	**	812.3	±	50.1	**	152.0	±	16.5	450.6	±	40.6
WT	32.1	±	3.8		291.1	±	18.2		219.0	±	29.7	513.9	±	8.0
A512T	30.1	±	4.3		294.0	±	41.4		223.2	±	29.1	504.9	±	64.7
M235A	19.5	±	1.6	**	321.2	±	38.4		152.7	±	34.8	514.7	±	43.1
M235C	19.9	±	5.1	**	382.5	±	52.8		158.3	±	15.7	528.9	±	38.1
A232C+M235A	21.1	±	1.9	*	314.2	±	18.5		148.0	±	18.3	773.8	±	260.3
A232S+M235A	18.7	±	1.5	**	317.7	±	58.2		151.7	±	24.4	558.8	±	57.6
A232S+M235C	16.2	±	2.5	**	218.3	±	29.3		190.0	±	28.9	630.4	±	87.5

The metal content of the yeast was measured using ICP-OES. The mean and standard deviation (SD) are shown (N=3) with the statistical evaluation compared to the WT using Dunnett's test (*$p<0.05$, ** $p<0.01$). This test was conducted using BY4741 in the presence of 20 μM $MnSO_4$, $CdSO_4$, $FeSO_4$, $CuSO_4$, $ZnSO_4$, and $CoSO_4$.

Supplementary Table 4. Properties of predicted transport pathways and metal-binding site

Plasmid	Tunnel length [Å]	Tunnel minimum radius [Å]	Hydropathy around metal-binding site
WT	**11.78**	**1.43**	**-0.30**
Q337K	10.71	1.15	-0.34
A512T	**11.10**	1.17	-0.42
M235A	**12.62**	**1.30**	**-0.16**
M235C	**13.05**	**1.45**	**-0.17**
M235D	8.19	**1.38**	0.33
M235E	**12.10**	**1.40**	-0.01
M235F	17.48	0.93	-0.55
M235G	**11.79**	1.21	0.04
M235H	8.66	**1.34**	0.24
M235I	**12.27**	1.23	**-0.15**
M235K	16.83	0.94	0.33
M235L	**12.14**	1.50	**-0.03**
M235N	9.13	**1.37**	0.15
M235P	10.18	**1.26**	**-0.08**
M235Q	9.32	**1.24**	0.24
M235R	-	-	-
M235S	**12.06**	**1.44**	0.07
M235T	**12.26**	1.48	-0.01
M235V	**12.51**	1.14	**-0.16**
M235W	21.82	1.02	-0.78
M235Y	21.23	0.96	-0.43
A232C	7.44	1.09	**-0.20**
A232M	**12.12**	1.01	0.01
A232S	**12.70**	**1.38**	0.01
A232V	10.69	1.17	-0.54
A232C+M235A	**10.88**	**1.24**	**-0.14**
A232M+M235A	14.10	0.93	**-0.13**
A232S+M235A	**13.61**	**1.43**	**-0.03**
A232V+M235A	14.07	1.15	**-0.19**
A232C+M235C	14.08	**1.43**	**-0.29**
A232M+M235C	10.24	1.02	0.04
A232S+M235C	**12.51**	**1.46**	**-0.03**
A232V+M235C	**13.15**	1.06	-0.31
Requirement	10.88 ~ 13.61	1.24 ~ 1.46	-0.30 ~ -0.03

The value within the requirement range for high Mn uptake efficiency (>60% in Tables 1 and 2) is highlighted by bold.
